# Pathophysiological aspects of transferrin-A potential nano-based drug delivery signaling molecule in therapeutic target for varied diseases

**DOI:** 10.3389/fphar.2024.1342181

**Published:** 2024-03-04

**Authors:** Chang Li, Liya Zhou, Xunzhe Yin

**Affiliations:** ^1^ Basic Medical College, Changchun University of Traditional Chinese Medicine, Changchun, China; ^2^ State Key Laboratory of Electroanalytical Chemistry, Changchun Institute of Applied Chemistry, Chinese Academy of Sciences, Changchun, China

**Keywords:** transferrin, transferrin receptor, targeted therapy, drug delivery system, iron

## Abstract

Transferrin (Tf), widely known for its role as an iron-binding protein, exemplifies multitasking in biological processes. The role of Tf in iron metabolism involves both the uptake of iron from Tf by various cells, as well as the endocytosis mediated by the complex of Tf and the transferrin receptor (TfR). The direct conjugation of the therapeutic compound and immunotoxin studies using Tf peptide or anti-Tf receptor antibodies as targeting moieties aims to prolong drug circulation time and augment efficient cellular drug uptake, diminish systemic toxicity, traverse the blood-brain barrier, restrict systemic exposure, overcome multidrug resistance, and enhance therapeutic efficacy with disease specificity. This review primarily discusses the various biological actions of Tf, as well as the development of Tf-targeted nano-based drug delivery systems. The goal is to establish the use of Tf as a disease-targeting component, accentuating the potential therapeutic applications of this protein.

## 1 Introduction

With the continuous innovation in drug development, an increasing number of novel medications are emerging for addressing a range of diseases. However, during the course of pharmaceutical development, challenges persist with three major aspects, namely, the unpredictable drug release rates, specificity in targeting tissues and cells and the stability of drugs. Additionally, issues related to systemic administration, such as potential toxic side effects induced by high doses, continue to be a concern (Sanjay et al., 2018). One strategy for improving the therapeutic index of medications involves utilizing drug delivery systems (Vyas et al., 2001). In contrast to conventional drug formulations, this innovative drug delivery system improves the stability of drugs within the body, delivers drugs specifically to targeted cells, confines the therapeutic effect to the site of pathology thus keeping healthy cells away from drug toxicity. Additionally, it can to some extent improve the solubility of drugs and protect them from degradation and oxidation (Rajput and Agrawal, 2010; Hossain et al., 2015).

Transferrin (Tf), as a naturally occurring protein in the body, has garnered significant interest in the realms of drug targeting and delivery systems, owing to its non-toxic, non-immunogenic, and biodegradable benefits. Simultaneously, the cell surface contains a significant quantity of transferrin receptors (TfR), which can achieve site-specific targeting in conjunction with Tf. Cells leverage the potential of the drug delivery system by transporting anticancer drugs and therapeutic genes through the Tf pathway into malignant proliferating cells that overexpress TfR (Kircheis et al., 2002).

This review provides a comprehensive summary of the structure, function, and clinical applications of Tf, focusing on utilizing of Tf and TfR in drug delivery systems, facilitating the transport of therapeutic agents to malignant tissues or cells.

## 2 Transferrin occurrence

Tf is believed to have originated in the evolution of vertebrates or early chordates, being one of the members of the Tf family. It was first isolated by Schade and Caroline (1946). It is primarily composed of hepatocytes (Beutler et al., 2000), with a molecular weight approximately around 80 kDa. Functioning as a vital chelator, it plays a crucial role in transporting iron within the serum (Huebers and Finch, 1987). Other cells and tissues that synthesize Tf include Sertoli cells (Lécureuil et al., 2004), ependymal cells (Tsutsumi et al., 1989), oligodendroglial cells (Bloch et al., 1985), and human breast cancer cell lines (Inoue et al., 1993). The concentration of Tf in the bloodstream is roughly 35 μM (approximately 2.5 mg/mL) (Parkkinen et al., 2002), with 30% of it binding to iron ions in the plasma (Leibman and Aisen, 1979).

The Tf family includes serum Tf, ovotransferrin (OTf), lactoferrin (Lf), melanotransferrin (MTf), and the cellular analogue, ferric ion binding protein (FBP). Tf in serum is found in diverse bodily fluids, including plasma, bile, amniotic fluid, cerebrospinal fluid, and milk (Qian et al., 2002). OTf is present in the oviduct secretions of birds and reptiles (Williams et al., 1982). Lf is present in milk, tears, and saliva (Metzboutigue et al., 1984). MTf is present on the cell surface and is associated with growth and differentiation (Sun et al., 1999). A recently discovered member of the Tf family, MTf (also referred to as p97), has been recognized as a crucial membrane protein in human malignant melanoma cells and certain fetal tissues (Rose et al., 1986). The concentration of Tf in plasma remains stable from birth, around 2–3 g/L, and it has a half-life of 8 days in the body (Campenhout et al., 2003). Tf is closely associated with human physiological health, and a concentration below 0.1 g/L is linked to an elevated risk of infection, growth retardation, and anemia (Hayashi et al., 1993).

## 3 Structural features

Tf is a monomeric glycoprotein composed of approximately 700 amino acids, playing a pivotal role in the body’s iron transport and storage. Tf binds with iron ions to form diferric Tf, aids in maintaining the body’s iron balance to meet its essential mineral needs. In red blood cells, the transport and release of iron are also associated with Tf, playing a vital role in the transport and distribution of oxygen.

Tf exhibits high sequence homology across different species and among various family members. For example, the homology between serum Tf in rabbits and humans is approximately 78%, while the homology between serum Tf and Lf is about 60% (Penezic et al., 2017). The high degree of conservation in the primary structure of Tf is dictated by its three-dimensional structure. Tf gains stability from 19 intrachain disulfide bonds, and Tf is safeguarded by three carbohydrate side chains, of which two being N-linked (Asn-413 and Asn-611), and the third being O-linked (Ser-32).

**FIGURE 1:**
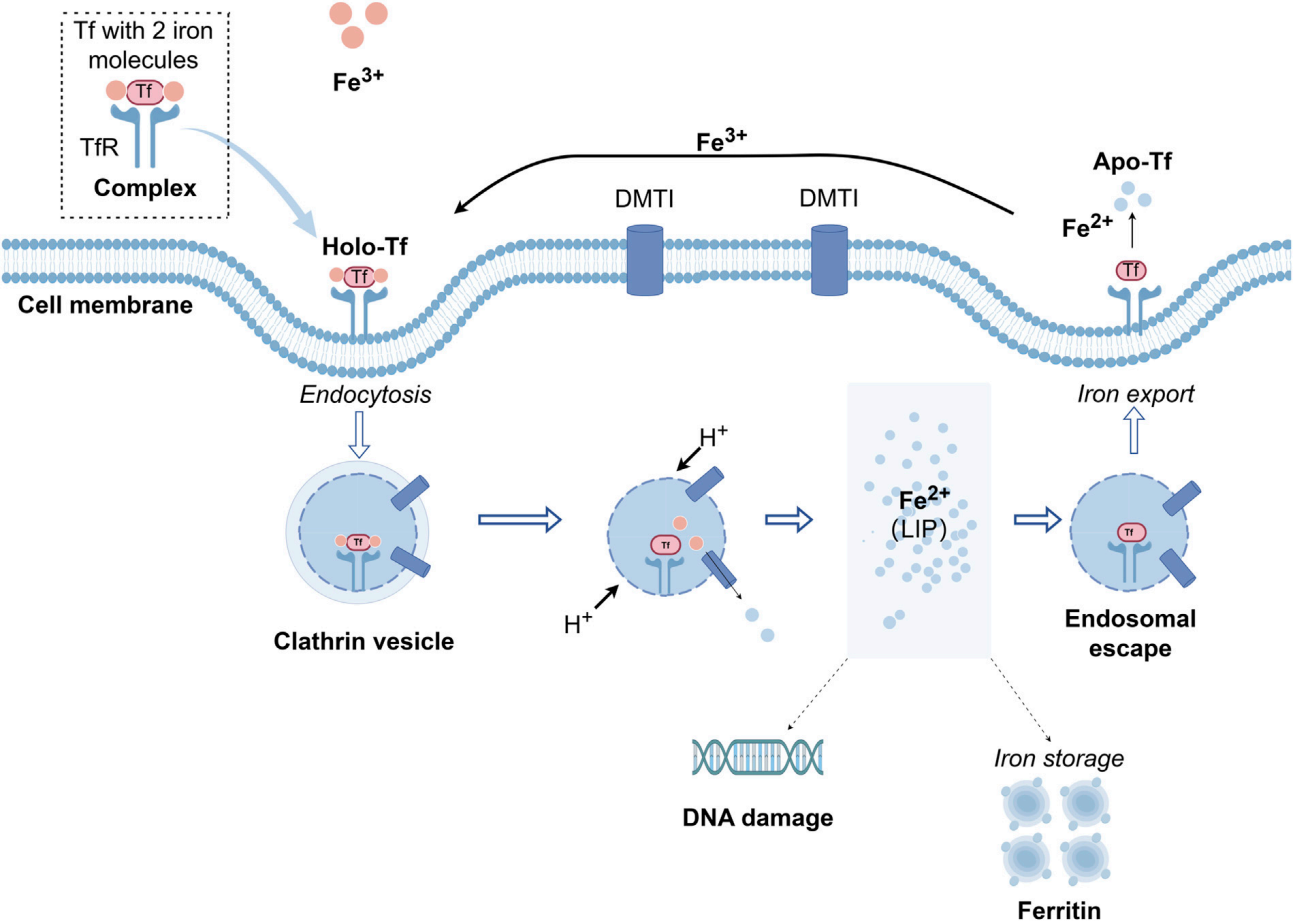
The cycle of cell iron uptake through the transferrin-transferrin receptor pathway. Differic transferrin (holo-Tf) binds to the TfR1 on the cell surface. These complexes position themselves within clathrin-coated pits, and the invagination is initiated and acidified through the action of a proton pump during endocytosis. During the acidification process of the endosome, iron is released from transferrin and transported out of endosomes through the divalent metal transporter DMT1. The apo-transferrin (apo-Tf) and TfR1 complex is then recycled through exocytic vesicles back to the cell surface and apo-transferrin is released into the circulation and re-used. Iron is stored as ferritin and hemosiderin. Tf, Transferrin; TfR1, Transferrin receptor 1; DMT1, divalent metal transporter.

The polypeptide chain of the Tf molecule folds into two evolutionarily related but functionally distinct lobes, namely, the N-lobe (336 amino acids) and the C-lobe (343 amino acids). A brief inter-lobe region connects these lobes through a short peptide sequence. Each lobe comprises two structural domains, each structural domain consisting of a series of α-helical domains that cover the central β-sheet scaffold. The interactions between these domains create a deep, hydrophilic cleft housing a binding site for Fe^3+^. Both the N-lobe and C-lobe also contain four conserved amino acid binding sites, consisting of 2 histidines, 1 aspartate, and 1 tyrosine. Tf without bound iron is termed apotransferrin (apo-Tf), while Tf that has already bound to iron is called holotransferrin (holo-Tf). At the metal binding site, Fe^3+^ is arranged in a distorted octahedral geometry (MacGillivray et al., 1998). Additionally, stability of Fe^3+^ is ensured by coordination with two oxygen atoms derived from carbonate molecules (Hirose, 2000). The surrounding amino acid residues are believed to enhance the stabilization of the metal binding site, ensuring that the structural domains adopt an open conformation crucial for the release of iron ions (He et al., 2000). Each molecule of Tf has the capacity to transport two trivalent iron ions (Fe^3+^) by interacting with TfR1 on the cell surface (Figure 1). The created Fe^3+^-Tf-TfR1 complex penetrates the cell via endocytosis mediated by membrane proteins (Qian et al., 2002; AlSawaftah et al., 2021). Apart from Fe^3+^, a variety of metal ions can also attach to the metal binding site. Tf participates in the transportation of various metal ions (He et al., 2000; Zhong et al., 2002).

## 4 Biological function

### 4.1 Tf-binds with iron

**FIGURE 2:**
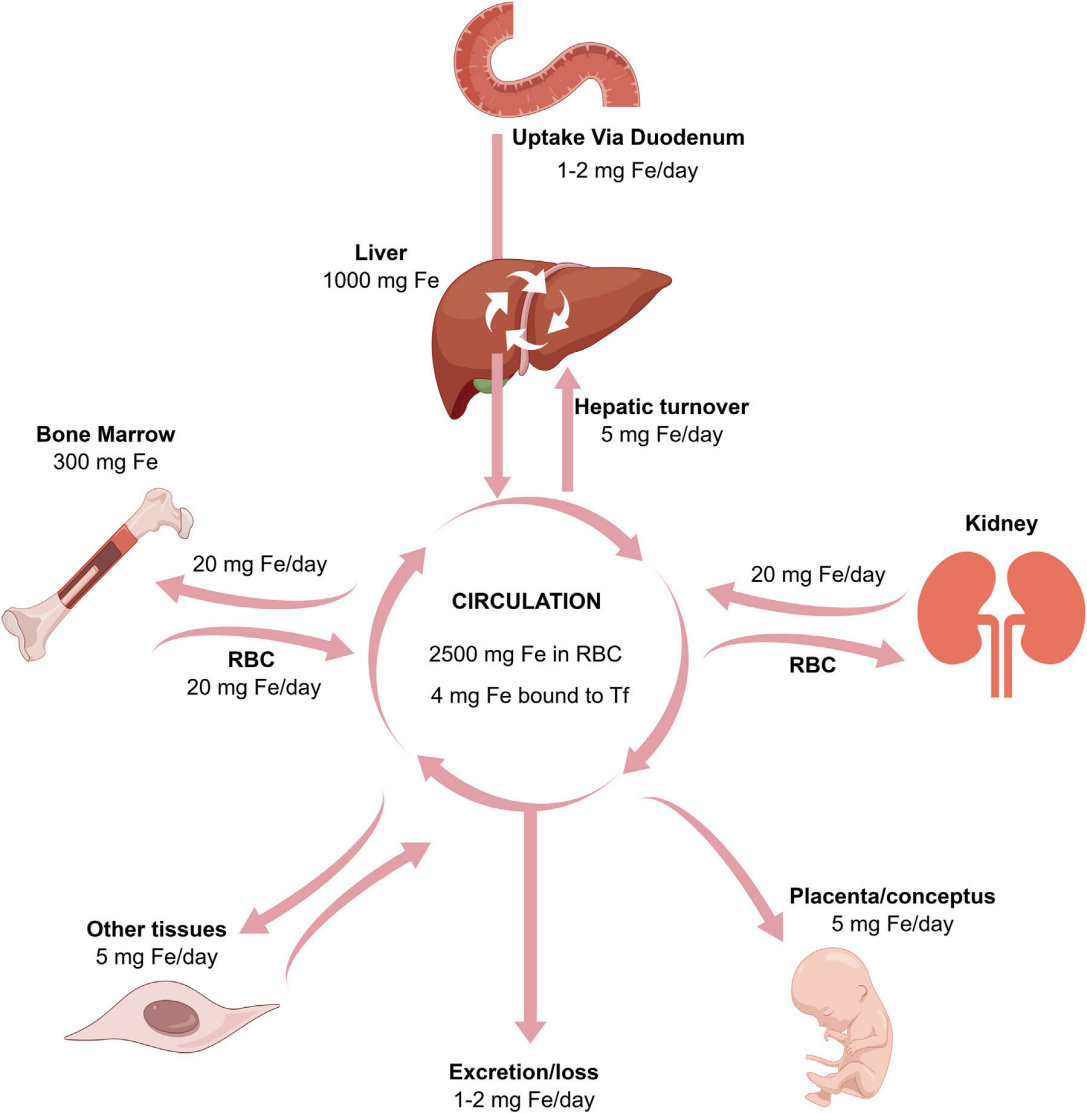
The relationships between the absorption, excretion, storage, placental, and bone marrow transport of iron.

Iron is one of the essential metallic elements in life, playing a crucial role in maintaining normal physiological activities. Various physiological and biochemical processes in the body are regulated by iron ions. Iron ions serve as cofactors for ribonucleotide reductase, participating in DNA replication, and are also engaged in the production of hemoglobin, cytochromes, and various enzymes (Li and Qian, 2002) (Figure 2). However, an excess of iron can generate free radicals via the Fenton and Haber-Weiss reactions, promoting oxidative stress and initiating lipid peroxidation, DNA damage, and other consequences, ultimately resulting in oxidative damage to tissues (Zhang et al., 2014; Kapitulnik, 2015). Both iron deficiency and overload can impact overall health (Crawford et al., 1998; Muckenthaler et al., 2017). The intricate regulation of iron homeostasis in the body involves overseeing the absorption, transport, utilization, and storage of iron. This intricate regulatory network involves the interaction of various proteins, with the most crucial one being Tf.

The primary biological functions of Tf are associated with its iron-binding properties. Tf not only transports iron ions in a form that is both soluble and non-toxic to various parts of the body for cell growth but also has the ability to remove free iron, providing a protective effect on the organism (Elsayed et al., 2016). The main route for iron ions to enter cells is through the interaction between Tf and TfR1. Tf binds to Fe^3+^ to form the Fe^3+^-Tf complex, which then gradually enters the cytoplasm by attaching to TfR1 situated on the cell surface to form a complex. The binding and release of iron by Tf are under the regulation of various factors, including pH, temperature, chelating agents, and ion concentration. Studies indicate that the stability of iron binding sites is maintained with the involvement of various anions, such as chloride ions. Under neutral conditions, chloride ions slow down the release of iron, while under acidic conditions, chloride ions can accelerate the release of iron. Adenosine triphosphate (ATP) can pump H^+^ ions into the cell through a proton pump, reducing the pH to 5.5. This weakens the binding between iron ions and Tf, thereby promoting the liberation of iron ions (Paterson et al., 1984). Upon entering the cell, Fe^3+^ undergoes reduction to Fe^2+^ facilitated by the metal reductase Six-Transmembrane Epithelial Antigen of the Prostate 3 (STEAP3), and subsequently, it is transported to the cytoplasm. Within the cytoplasm, a portion of Fe^2+^ participates in the biosynthesis of specific target proteins, and concurrently, another portion is sequestered in ferritin to avert the accumulation of excess free iron ions, which could lead to oxidative stress. This stored iron can be released when needed, undergoing degradation in lysosomes. After releasing iron ions, Tf become capable to create a complex with TfR1 and return to the cell surface, subsequently re-entering the bloodstream. This cycle continues until it binds to iron ions again, forming a complex, and the process repeats. Each Tf molecule can complete this cycle approximately 100 times (Abdizadeh et al., 2017).

Serum Tf is regarded as a component of the overall metal transport system in the human body, it can uptake iron ions and transport them to cells and tissues through the systemic circulation (Baker and Morgan, 1969). In addition to iron, it can also be involved in transporting various other metal ions, including therapeutic metal ions (Savigni and Morgan, 1998), radiodiagnostic metal ions, some toxic metal ions, as well as other widespread metal ions such as Mn^3+^, Ga^3+^, Ti^4+^, and Hf^4+^ (Sun et al., 1999). Hemopexin exhibits a less pronounced role in iron uptake; instead, it might promote accelerated proliferation of tumor cells by acting as an iron scavenger on the cell surface to hinder lipid peroxidation (Kwok and Richardson, 2002).

### 4.2 Tf acts as an antimicrobial activity

The Tf protein exhibits antimicrobial activity. Studies have found that the proliferation and invasion of various pathogenic microorganisms depend on iron elements. Elevated levels of free iron in the body foster the proliferation of pathogenic microorganisms (Teehan et al., 2004). Inhibiting the uptake of iron by pathogenic microorganisms is a primitive host defense strategy. Through the utilization of Tf to sequester free iron, the growth of diverse pathogenic microorganisms, such as Gram-positive bacteria (*Staphylococcus aureus*), Gram-negative bacteria (*Pseudomonas aeruginosa*), and fungi (*Candida* albicans), can be hindered (Lin et al., 2014; Bruhn and Spellberg, 2015).

In clinical settings, diseases that lead to elevated levels of free iron, such as hereditary and acquired hemochromatosis, liver failure, congenital heart surgery, and hematologic malignancies, usually associate with a higher occurrence of bacterial infections (Beutler et al., 2003). Research has revealed that apotransferrin can reduce infections by sequestering free iron (von Bonsdorff et al., 2003). At the same time, it can reduce the adhesive capabilities of both Gram-negative and Gram-positive bacteria to surfaces (Ardehali et al., 2003). OTf and Lf are considered primary antimicrobial agents. They function by chelating iron ions necessary for microbial growth, thereby controlling the availability of iron ions and inhibiting microbial activity (Iyer and Lonnerdal, 1993; Lonnerdal and Iyer, 1995).

### 4.3 Tf acts as growth factor and cytoprotection

Tf is involved in various activities such as growth and differentiation, including muscle growth (Shimooka et al., 1986), embryonic development (Ohtsuka et al., 2001), cell mitosis (Sirbasku et al., 1991), and angiogenic activities (Cancedda et al., 1997). Research has found that differences in developmental stages or physiological states can affect the regulatory function of Tf in cell growth and development. Through intracerebral injection of lipo-Tf, phenomenon of oligodendrocyte precursor cells undergo rapid differentiation was observed in 2-7-day-old rats, whereas this phenomenon was not observed in 10-day-old rats (García et al., 2003). Similarly, Tf is linked to the proliferation of human colorectal tumor cell lines. For the less differentiated HCT116 cells, Tf leads to an increased binding of its epidermal growth factor (EGF) to its receptor, while more differentiated colorectal cancer cell lines exhibit the opposite effect (Zirvi, 1991; Yeoman et al., 1996). The combination of iron with ferritin has been shown to impede apoptosis in ovarian cancer cells. Signal molecules involved in the apoptosis pathway (Myc, FasL, TNF-α, and TRAIL) might have a pivotal role in regulating cell survival and death by adjusting intracellular iron levels through the upregulation of ferritin (Fassl et al., 2003). In addition, Tumor cells can restore intracellular iron levels and prevent cell death by binding iron to further ferritin. Ferritin, to some extent, can offer protection to lymphohematopoietic cells and hepatocytes, shielding them from damage caused by Fas-mediated cell death mechanisms (Lesnikov et al., 2001).

The dual functions of Tf, acting both paracrinely and autocrinely, have been demonstrated. Hypertrophic chondrocytes have the capability to produce a substantial quantity of Tf, which can be released into the surrounding environment through paracrine secretion. This enables undifferentiated cells to interact with Tf released by hypertrophic chondrocytes, utilizing TfR1 on their surface (Gentili et al., 1994; Menter et al., 1995). Studies have uncovered that when Tf binds to insulin-like growth factor binding protein 3 (IGFBP-3), it loses its capacity to stimulate bladder smooth muscle proliferation and induce apoptosis in prostate cancer cells (Weinzimer et al., 2001). Tf is considered an antimicrobial agent, inhibiting microbial antimicrobial activity by chelating iron ions on one hand. On the other hand, irrespective of its iron-binding properties, Tf functions as a growth factor, participating in the modulation of immune and inflammatory responses (Lonnerdal and Iyer, 1995).

## 5 Clinical applications of TF

The multiple actions of Tf can be exploited to produce a range of potential therapeutic applications (Table 1).

**TABLE 1:** Potential therapeutic applications of transferrin.

Potential therapeutic applications of transferrin			
Pathophysiological condition	Clinical applications	Therapeutic options	References
Transferrin levels deficiency	Atransferrinemia	Plasma transfusion	Trombini et al. (2007)
		Transferrin replacement therapy	Beutler et al. (2000)
		Iron chelators	Adams and Barton (2011)
Free iron and/or iron overload	Ischemia reperfusion injury	Regulate the expression of transferrin to inhibit Fe^2+^	Gao et al. (1996), Perez et al. (2009)
		Use apo-Tf to clear free iron	Hernandez et al. (1987)
		Anti-oxidative	Kaminski et al. (2002)
Growth and differentiation	Tumor or cancer	Targeted drug delivery via transferrin-transferrin receptor pathway	Li and Qian (2002)
		Promote cytotoxicity and proliferation of natural killer cells	Okamoto et al. (1996)

### 5.1 Atransferrinemia

Atransferrinemia is an uncommon genetic disorder resulting from irregularities in iron metabolism, and it is inherited as an autosomal recessive trait (Hayashi et al., 1993; Beutler et al., 2000). Cases of atransferrinemia are extremely rare, with fewer than 15 reported worldwide to date. This condition exhibits a unique characteristic of being unrelated to race or gender prevalence, and it predominantly manifests in individuals aged 1–2 years. The clinical presentation includes severe microcytic anemia, and without prompt treatment, there is a risk of stunted growth, serious complications related to iron and, in extreme cases, can lead to death. From a biochemical standpoint, there is a significant deficiency in the levels of Tf in the serum. The shortage of serum Tf results in the impairment of its iron clearance and transport functions, causing profound iron-deficiency anemia in the body and substantial iron overload in non-hematopoietic tissues. Hypotransferrinemic (hpx/hpx) mice function as a model for comprehending this disorder (Trenor et al., 2000). The severe anemia observed in hpx/hpx mice and patients with hereditary atransferrinemia indicates that when the Tf-Fe^2+^-transferrin receptor 1 (TfR1) cycle pathway is unable to function properly, it results in a reduction of iron entering erythroid precursors (Bernstein, 1987).

The treatment of atransferrinemia can be carried out through plasma transfusion. The suggested transfusion regimen includes plasma transfusions every 2 weeks during the initial anemic stage and every 4 weeks in the maintenance phase. The transfusion volume is tailored according to the patient’s weight to attain a post-transfusional Tf level of 50–60 mg/dL. Infusing lipoprotein-Tf complexes can also be used to treat this condition. By administering injections of 1–2 g of pure lipoprotein-Tf every 3–4 months over a period of 4–7 years, it can reduce the impact of growth delay and other associated effects of the disease (Hayashi et al., 1993).

### 5.2 Ischemia reperfusion injury

Ischemia-Reperfusion (IR) injury pertains to the clinical syndrome in which, upon the restoration of blood flow after a period of ischemia (lack of blood supply), cells in ischemic tissues undergo more severe damage to their structure and metabolism compared to the damage incurred during the ischemic phase. This results in an exacerbation of tissue injury and a further deterioration of organ function (Conrad et al., 2016). Diseases such as stroke (Schaller and Graf, 2004), cardiovascular disorders (Wernly, 2004), renal failure (Lien et al., 2003), and organ transplantation (Ohkohchi et al., 1999) are all associated with IR injury (Kalogeris et al., 2012). Iron ions, serving as crucial mediators in the electron transport chain, play a key role in mitochondrial oxidation reactions and oxygen transport. After tissue ischemia, mitochondrial dysfunction results in the production of a substantial quantity of reactive oxygen species, causing changes in the ion permeability of cell membranes. Consequently, a substantial release of unstable iron ions occurs, and the increased levels of free iron ions further catalyze the production of free radicals. This cascade of events results in lipid peroxidation and ferroptosis, exacerbating ischemic damage within the impacted tissue (Stockwell et al., 2017; He et al., 2020).

Studies have uncovered differing degrees of increase in the levels of iron ions, Tf, and TfR1 in the brains of individuals with ischemic stroke (Ingrassia et al., 2019). Utilizing iron chelators to eliminate iron demonstrates a protective effect against IR injury (De Vries et al., 2004). Baicalin, as a natural inhibitor of ferroptosis, can regulate the expression of Tf, inhibit Fe^2+^, and suppress lipid peroxidation of cell membranes, thereby preventing cell death (Yang et al., 2021). Further research has shown that baicalin can also act as an iron chelator by forming iron-baicalin complexes. This role helps regulate iron ion balance and inhibit the Fenton reaction (Gao et al., 1996; Perez et al., 2009).

A potential therapeutic strategy involves utilizing apo-Tf to eliminate free iron participating in redox reactions. In animal models of IR injury, the intraperitoneal injection of apo-Tf has been demonstrated to decrease the formation of circulating redox-active iron and superoxide. This exerts a mitigating effect on renal IR injury in mice. In an animal model of intestinal IR injury, intravenous administration of apo-Tf can alleviate intestinal vascular permeability (Hernandez et al., 1987).

### 5.3 Cancer therapy

Unrestrained cell division and evasion of the immune system to avoid cell death are among the pivotal characteristics of tumor cells (Hanahan and Weinberg, 2011). Numerous studies propose that cancer cells necessitate a higher amount of iron than normal cells to facilitate their rapid growth and proliferation. Cellular carcinogenesis can disrupt iron metabolism, causing the abnormal expression of iron-related proteins such as Tf within tumor cells (Yu et al., 2017). The close association between iron metabolism and the elimination of tumor cells, along with the inhibition of tumor growth, have together positioned Tf as a potential novel target for cancer treatment.

Studies have suggested that the lipoprotein Tf holds promise for cancer treatment. The collaborative effect of Tf, insulin-like growth factor-1 (IGF-1), and interleukin-2 (IL-2) can more efficiently stimulate certain immune cell types, such as lymphokine-activated killer cells (LAK) and natural killer cells (NK), augmenting their cytotoxicity and proliferative capacities. This contributes to a more efficient immune response against potential threats. Furthermore, a dedicated cell growth medium containing Tf, referred to as RDSF, has the capacity to stimulate the growth and proliferation of LAK cells (Okamoto et al., 1996). The co-administration of Tf and the antimalarial drug artemisinin (ART) can enhance the drug resistance in small cell lung cancer (SCLC). *In vitro* studies demonstrate that after Tf pre-treatment, ART is able to eliminate SCLC cells at nanomolar concentrations (Sadava et al., 2002).

## 6 Transferrin-transferrin receptor system

TfR1 are ubiquitously found on the cell membrane surface and constitute a transmembrane glycoprotein. It is created through the cross-linking of two subunits by disulfide bonds, with each subunit having a molecular weight of 90 kDa, resulting in a homodimeric structure (Aisen, 2004). The gene encoding TfR1 is situated in the q21-25 region of chromosome 3. It is mRNA expression is under the regulation of iron, and its role encompasses facilitating the cellular uptake of iron by binding with iron-loaded Tf (Gammella et al., 2017). TfR1 preferentially binds to Tf molecules carrying two iron ions, forming a Fe-Tf-TfR1 complex, which enters the cell through endocytosis. During endocytosis, the complex is enveloped by clathrin, forming an endocytic vesicle. Within the vesicle, proton pumps utilize energy provided by ATP to pump H^+^ into the vesicle, lowering its pH to 5.5 or below. This acidic environment induces a conformational change in Tf, releasing iron ions into the cytoplasm. The iron ions are then stored in the ferritin in their divalent form (Gkouvatsos et al., 2012). Simultaneously, the Tf-TfR1 complex is recycled to the cell surface via exocytosis. Subsequently, as the extracellular fluid pH increases, the dissociation occurs between Tf and TfR1. Tf returns to the bloodstream, mediating the next round of transport. The entire cycling process takes only 10–20 min (Hopkins and Trowbridge, 1983).

There are two recognized types of TfR, TfR1 and transferrin receptor 2 (TfR2). Among these, TfR1, also referred to as CD71, stands out as the predominant subtype. It is expressed in almost all cells of the human body. Its conformation and affinity for Tf can undergo alterations depending on the body’s pH, playing a vital role in facilitating the absorption of iron bound to Tf into the cells (Kawabata et al., 1999). TfR2 is the second subtype of TfR1, and it shares a similar structural composition with TfR1. TfR2 is frequently found in hepatocytes and plays a role in overseeing and preserving the absorption and distribution of iron ions in the body through the modulation of hepcidin hormone levels. This helps maintain dynamic balance in iron ion levels within the body (Kawabata et al., 2000).

Research indicates that compared to normal cells, various cancer cell lines such as pancreatic, colorectal, lung, and bladder cancers exhibit significantly elevated levels of TfR1 (Ryschich et al., 2004; Prutki et al., 2006). Moreover, the degree of TfR1 expression shows a positive correlation with the malignancy of tumors. This is presumably to fulfill the heightened demand for iron required by cancer cells for their growth and proliferation (Singh et al., 2011). Studies suggest that TfR1 has the capability to activate the NF-κB signaling pathway through its interaction with IKK (Inhibitor of the NF-κB Kinase). This interferes the balance between cell proliferation and apoptosis, promoting tumor cell survival by inhibiting apoptosis (Kenneth et al., 2013). Additionally, TfR1 can induce the generation of reactive oxygen species by promoting mitochondrial respiration. This oxidative stress can lead to DNA mutations, contributing to the occurrence and metastasis of tumors (Daniels-Wells and Penichet, 2016). Diminishing the levels of TfR1 expression and suppressing the mRNA expression of TfR1 can effectively hinder the proliferation of tumor cells. Research indicates that TfR1 is involved in cellular immune regulation. TfR1 is highly expressed on the glomerular mesangium, and it plays a role in immune regulation by binding with IgA in the glomerular mesangial cells. This involvement in immune regulation extends to the processes of apoptosis and proliferation in cells (Berthelot et al., 2012).

## 7 Transferrin/transferrin receptor mediated drug delivery

**FIGURE 3:**
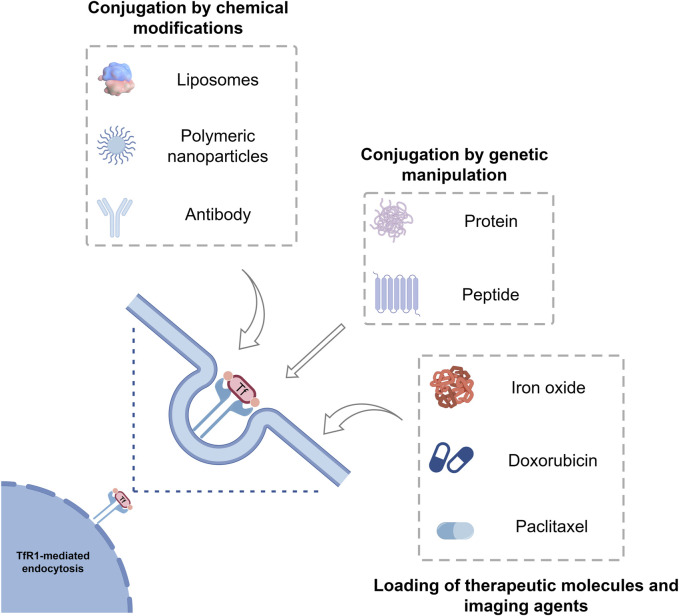
Tf as a protein-based delivery system for oncological therapeutics and imaging agents. Tf can be loaded with different drugs, imaging agents, liposomes, polymeric nanoparticles, inorganic nanoparticles and have intrinsic targeting capabilities toward the receptor TfR1, which is overexpressed in many tumors. Tf, Transferrin; TfR1, Transferrin receptor 1.

One of the strategies to reduce the adverse effects of drugs and enhance therapeutic efficiency is to specifically deliver the drugs to target cells, for example, through methods such as nanoparticles (NPs), liposomes, or targeted drug carriers (Table 2). Drug delivery systems possess the potential to extend the circulation duration of drugs in the body, enhance drug metabolism patterns, and regulate the distribution of drugs within the body. Therefore, Drug delivery systems mediated by ligand-receptor interactions have garnered significant attention (Vyas et al., 2001; Barratt, 2013). Natural ligands and their receptors present in the human body not only enable site-specific targeting but also offer advantages (Vyas and Sihorkar, 2000). Tf and its receptor, serving as targeting ligands, can deliver therapeutic drugs to malignant sites overexpressing the TfR1. Drug delivery systems, as a novel therapeutic approach, have vast prospects. Studies suggest that, in comparison to the development of new drugs, the creation of innovative drug delivery systems offers the benefits of a shorter development period and reduced costs (Zhang et al., 2013) (Figure 3).

**TABLE 2:** Examples of transferrin-conjugated and -targeted nano-based drug delivery systems.

Direct conjugation of transferrin receptor to anticancer drugs			
Conjugated compound	Targeting moiety	Study model	References
Saporin	Chimeric human TfR antibody genetically fused to avidin (IgG3)	U266 myeloid/plasmacytoma lymphoblast cell lines	Daniels et al. (2007)
	Human Tf	GL-15 human glioblastoma multiform cell lines	Cimini et al. (2012)
Diphtheria toxin	Anti-human TfR antibody (IgG1)	M21 cell lines	Trowbridge and Domingo (1981)
	Human Tf (CRM-107)	Phase I and II clinical trials	Weaver and Laske (2003)
Doxorubicin	Transferrin-conjugated biodegradable polymersome	C6 cell line	Pang et al. (2011)
	Transferrin conjugated magnetic silica PLGA	U87MG tumor-bearing mice	Cui et al. (2013)
Cisplatin	Covalently linked with gallium (Ga-Tf)	Mcf-7 and HeLa human cervical cancer cell lines	Head et al. (1997)
	Tf-PEG-liposome	MKN45P, tumor bearing mice	Iinuma et al. (2002)
Paclitaxel	Tf-conjugated nanoparticle	PC3 human prostate cancer cell line	Sahoo et al. (2004)
Delivery through the blood brain barrier for the treatment of brain glioma			
Paclitaxel	Tf-conjugated nanoparticles	C6 rat glioma cell line	Shah et al. (2009)
Resveratrol	Lactoferrin	C6 cell line	Guo et al. (2013)
Docetaxel	Tf-conjugated nanoparticles	Male Sprague-Dawley rats inoculated with C6 cell	Gan and Feng (2010), Guo et al. (2013)
Transferrin-conjugated nanoparticles as contrast agents for imaging applications			
Gadolinium-based contrast agent (Gd-DTPA)	SiO_2_-coated quantum dots (QD)	Male mice	Korkusuz et al. (2012)
Gadolinium-based contrast agent (Magnevist)	Tf-conjugated liposomes	Athymic nude mice inoculated with PC-3M-Luc cells	Korotcov et al. (2010)
Doxorubicin	Tf-conjugated nanoparticles	HeLa cell line	Chen et al. (2012)

### 7.1 Direct conjugation of transferrin receptor to anticancer drugs

Cancer has emerged as one of the primary causes of mortality globally. Currently, traditional cancer treatment methods such as surgical resection, chemotherapy, and radiation therapy have drawbacks, including limited efficacy, susceptibility to drug resistance, and significant toxic side effects (Xia et al., 2022). Targeted therapy has the potential to extend drug circulation time, reduce systemic toxicity, and precisely deliver therapeutic compounds to the site of the disease. Therefore, it is of significant importance to search for, develop, and utilize a stable tumor marker for targeted therapy in the treatment of tumors.

TfR1 is widely expressed in various tissues and cells throughout the human body, but its expression levels vary significantly based on the cellular proliferation and metabolic conditions. Studies have identified a high expression of TfR1 in numerous cancer cells, including pancreatic cancer, colorectal cancer, and lung cancer, in contrast to normal cells. The contrasting expression of TfR1 between tumor cells and normal cells represents a promising target for cancer therapy. Tf, antibodies, and nucleic acid aptamers are all ligands for TfR1. Constructing a targeted drug delivery system by loading chemotherapy drugs onto these ligands can achieve targeted treatment of tumors while reducing damage to normal cells. TfR1 is not only reusable, but the internalization cycle of Tf-TfR1 pathway is rapid and efficient, averaging only 10 min per cycle (Dautryvarsat et al., 1983). This allows for the rapid entry of drugs into cells (Liu et al., 2013a), enabling a short-term increase in drug concentration within the cells to exert its therapeutic effects (Chang et al., 2012; Nam et al., 2013). The Tf-TfR1 delivery system enables site-specific delivery of various therapeutic metal ions, drugs, proteins, and gene.

The covalently linked complex of Tf and doxorubicin (DOX) exhibits cytotoxic effects in various cancer cell lines, including breast cancer, cervical cancer, and liver cancer cell lines (Faulk et al., 1990; Singh et al., 1998). DOX as a common chemotherapy drug, is widely used in the treatment of malignant tumors such as lymphoma and leukemia. However, research has revealed that DOX lacks targeted effects and exhibits dose and time dependence. It can accumulate significantly in the heart, causing serious damage to the cardiac tissue (Carvalho et al., 2014). NPs are widely utilized in cancer treatment due to their excellent internalization, high stability, large drug-carrying capacity, and reduced accumulation in the liver (Chompoosor et al., 2010). Loading DOX onto NPs and then coupling it with Tf, leveraging the recognition of TfR1, allows for the specific transport of DOX to tumor cells. This targeted delivery approach enhances the therapeutic effectiveness of DOX in oncological therapy (Szwed et al., 2014). Moreover, by surface modification of DOX liposomes with Tf and folic acid, it effectively inhibits tumor growth, enhances mouse survival rates, and minimizes toxic side effects (Gao et al., 2013).

β-elemene is an efficient and low-toxicity anti-cancer active ingredient extracted and isolated from Curcuma *wenyujin*. However, β-elemene has drawbacks such as strong hydrophobicity and low bioavailability. Although the development of oral formulations and injectable solutions has improved its water solubility and bioavailability, clinical applications still face challenges like significant vein irritation, weak tumor-targeting capability, and insufficient therapeutic efficacy (Zhai et al., 2018). The study found that designing β-elemene as a Tf-modified microemulsion, which specifically binds to Tf receptors on lung cancer cells, demonstrated enhanced cytotoxicity and apoptosis. *In vivo* experiments, it was proven to achieve an inhibition rate of 80% on tumors, prolonging the survival period of mice, and exhibiting low systemic toxicity (Zhang et al., 2019).

The primary function of Tf lies in its ability to transport iron, a crucial regulator of cellular growth. Studies indicate that the presence of intracellular iron can activate ART (AS), leading to potent cytotoxic effects on tumor cells both *in vivo* and *in vitro*. Consequently, the research team led by Hou chose to anchor Tf as a targeting molecule onto the surface of copper sulfide nanoparticles, utilizing it for the delivery of ART. This system exhibits selective targeting of tumor cells, absorbed by breast cancer cells through Tf-mediated endocytosis. Simultaneously, it transports ART and iron ions to the tumor, thereby enhancing its anti-tumor activity (Hou et al., 2017). The observed efficiency of Tf in drug transport has been utilized by conjugating Tf with diphtheria toxin, resulting in cytotoxic effects in brain cancer cells. The administration of diphtheria toxin conjugated with Tf (TF-CRM107) has been injected into the tumors of patients with malignant brain tumors in clinical settings. Positive anti-tumor effects have been noted in patients with malignant brain tumors that exhibit resistance to conventional treatments (Wang et al., 2000; Weaver and Laske, 2003). Carmustine (BCNU) is the most commonly used chemotherapy drug for brain gliomas, but severe side effects occur when administered intravenously. The study found that the preparation of a novel TF-BCNU-PLGA nanoparticle system by modifying PLGA carriers with Tf protein can exert a stronger inhibitory effect on glioma cells (Chunsheng et al., 2007).

The chimeric human-mouse IgG3 biotin-fused antibody (ch128.1Av) possesses the ability to specifically recognize TfR1 (Daniels et al., 2007), reducing the expression levels of TfR1. When conjugated with saponin, this antibody can target and treat malignant B-cell lymphoma while minimizing toxic side effects (Daniels-Wells et al., 2013). The TfR1 monoclonal human anti-human IgG1 antibody, when conjugated with a radioactive substance, is capable of reducing the volume of pancreatic cancer tumors and extending the survival period of mice (Daniels-Wells et al., 2013; Sugyo et al., 2015). Tf protein, when dual-liganded with temozolomide, can be used for the treatment of glioblastoma (Kumar et al., 2007; Li et al., 2012). Similarly, other chemotherapy drugs such as cisplatin, paclitaxel, and the like, when covalently linked with Tf, can enhance the sensitivity of tumor cells.

### 7.2 Transferrin receptor mediated drug delivery through the blood brain barrier

Central nervous system (CNS) diseases encompass neurodegenerative disorders and brain tumors, such as Parkinson’s disease, Alzheimer’s disease, and gliomas. Over 1.5 billion people worldwide are affected by CNS diseases (Vieira and Gamarra, 2016). The occurrence and fatality rates of CNS disorders are increasing, propelled by the aging population and the accelerated pace of life. These conditions typically have a prolonged course and slow treatment progression. In the coming decades, more people are expected to be affected by Alzheimer’s, Parkinson’s, and cerebrovascular diseases.

Currently, the primary treatment methods for CNS diseases include systemic administration, intracranial injections, and brain implants. However, these approaches face limitations due to a lack of clear brain targeting, thereby restricting their effectiveness. The blood brain barrier (BBB), a dynamic physical barrier, is positioned between the bloodstream and brain tissue. It consists of tightly linked brain capillary endothelial cells (BCECs), pericytes, astrocytes, and neurons, creating a compact endothelial cell membrane (Bhowmik et al., 2015; Posadas et al., 2016). The BBB serves as a functional barrier stabilizing the internal environment of the CNS. It exhibits extremely stringent selectivity and restrictiveness for molecules entering the brain. While facilitating the entry of nutrients, it prevents harmful substances in the bloodstream from accessing the brain. The BBB additionally restricts the interchange of ions and fluids within the brain, virtually preventing 98% of small molecule drugs and nearly all large molecule drugs from entering brain tissues (Garg et al., 2015). The BBB acts as an obstacle to the intracranial delivery of therapeutic drugs for the nervous system. The BBB obstructs the delivery of therapeutic drugs to the brain for intracranial diseases, impeding the entry of many drugs. Even if a small quantity manages to enter, it frequently falls short of achieving effective therapeutic concentrations within the brain (Jones and Shusta, 2007). Therefore, the BBB has become a hurdle for the transport of therapeutic drugs to the brain in neurological treatments. Developing successful strategies to bypass the BBB is crucial for obtaining tools to treat the CNS.

In recent years, there has been a focus on biological therapies for CNS-related diseases. Several pharmaceutical and biotechnology companies have developed various antibody variants targeting β-amyloid plaques associated with Alzheimer’s disease, such as solanezumab (Doody et al., 2014), gantenerumab (Ostrowitzki et al., 2017), crenezumab (Cummings et al., 2018), and those targeting α-synuclein associated with Parkinson’s disease, such as prasinezumab (Jankovic et al., 2018) and BIIB054 (NCT03318523). These antibodies have consistently demonstrated therapeutic efficacy in preclinical models (Sevigny et al., 2016). However, after entering the third clinical trial, all were discontinued largely due to futility (Salloway et al., 2014; Logovinsky et al., 2016). The commonality among all failed clinical trials is that the accumulation of therapeutic antibodies in the brain relies on the passive mechanism of uptake across the blood-brain barrier.

Receptor-mediated transport is currently one of the most mature methods for drug delivery to the brain. Active targeted drug delivery has become a hot topic in modern research on brain-targeted drug delivery systems, leveraging specific receptors and transport mechanisms within the BBB. Research has found that, in contrast to endothelial cells in other organs, the BBB surface has endogenous antibodies capable of specific recognition, such as the common TfR. The selective expression of the TfR in brain capillaries may lead to the preferential accumulation of TfR-targeted substances in the brain (Friden et al., 1991). A promising strategy involves targeting CNS active drugs to interact with carrier and receptor proteins associated with the BBB. Research has identified one targeting approach that focuses on the TfR expressed by BCECs (Johnsen and Moos, 2016). The study found that attaching larger nanocarriers to different TfR-targeting molecules can increase the brain concentration of the encapsulated drug cargo (Zhang et al., 2004). Multiple studies have indicated that endogenous Tf, as a targeting molecule for liposome transport, has various therapeutic effects, including improvement after stroke and brain injury (Omori et al., 2003; Reddy et al., 2006), treatment of glioblastoma multiforme (GBM) tumors (Jhaveri et al., 2018; Lakkadwala and Singh, 2019), and gene silencing after siRNA delivery into the brain (Cardoso et al., 2010).

Rosemary acid, as a natural compound, possesses various pharmacological effects such as anti-inflammatory, antioxidant, and anticancer properties (Baek and Lee, 2016). Research has found that co-loading rosmarinic acid with solid lipidnano particles (SLN) and surface modification with Tf on SLN can enhance targeting to blood-brain barrier cells. This approach may reduce the toxicity of the drug to the blood-brain barrier, while also increasing antioxidant stress capabilities (Kuo et al., 2020).

Antibodies face limitations such as poor stability, strong immunogenicity, and large molecular size, which restrict their application. Peptide ligands are gaining more popularity as they can circumvent these issues. T7 is a peptide ligand exhibiting a strong affinity for TfR1. Using T7 as an effective brain-targeting ligand, targeted molecular modification of gene-loaded dendrimeric polymers can achieve gene therapy for brain tumors, significantly enhancing gene expression within the brain (Kuang et al., 2013). Research suggests that the T7 peptide can enhance the accumulation of shRNA in the brain, thereby mediating the downregulation of vascular endothelial growth factor (VEGF), with the aim of treating GBM (Kuang et al., 2016). Using T7 peptide-functionalized polyethylene glycol (PEG) -PLGA micelles, we constructed micelles conjugated with T7 peptide and loaded with BCNU. These micelles can selectively bind to the Tf overexpressed on the blood-brain barrier and glioma cells, primarily concentrating in the glioma while showing minimal accumulation in major organs (Bi et al., 2016; Wei et al., 2016). To enhance the performance of the targeted peptide, researchers developed a red blood cell membrane-coated solid lipid nanoparticle (RBCSLN). RBSCLN, based on natural lipid solid lipid NPs, was formed by wrapping erythrocyte membranes modified with T7 peptide to bind TfR1 and cross the BBB. *In vivo* studies demonstrated that this innovative design resulted in a 50% reduction in tumor growth (Fu et al., 2019). Research has revealed that T7 peptide-modified liposomes can serve as carriers for delivering HER2 inhibitors, actively targeting breast cancer tumors while concurrently reducing toxicity to normal tissues (Zhang et al., 2021). Simultaneously, T7 peptide-modified polymers exhibit efficient cellular uptake capabilities in breast cancer MCF-7 cells, along with rapid escape from endosomes/lysosomes (Gao et al., 2014).

### 7.3 Transferrin-conjugated nanoparticles as contrast agents for imaging applications

The progress in imaging technology carries substantial implications for the identification, detection, and treatment of diseases, enabling a relatively non-invasive, quantitative, and timely visualization of tissues, morphology, and function. Imaging requires a high level of sensitivity and specificity to unveil physiological and pathological changes at the cellular level (Jonas and Gutowsky, 1968). Magnetic Resonance Imaging (MRI), relying on the physical phenomenon of nuclear magnetic resonance, has become a commonly employed diagnostic method for conditions such as tumor detection, leveraging its high soft tissue resolution and non-ionizing characteristics. In 1990, precise modification of ultrasmall superparamagnetic iron oxide (USPIO) was accomplished by employing arabic gum (AG), facilitating its capture by sialic acid glycoprotein receptors on the surface of hepatocellular carcinoma cells. This enhanced the targeting of contrast agents to the liver, facilitating weighted imaging for liver cancer (Reimer et al., 1990). While MRI is widely utilized in clinical settings, it has been observed that some tissues exhibit minimal differences in magnetic resonance signal intensity. This challenge makes it difficult to distinguish between lesions and normal tissue on the images. Additionally, there are limitations such as short imaging times, rapid diffusion, lack of specificity, and the occurrence of adverse reactions in patients, which further restrict its usage (McAteer and Choudhury, 2009). The development of low-cost, minimally toxic, and highly functional novel contrast agents is a current research hotspot.

The inability to penetrate the blood-brain barrier is currently one of the limitations of clinically relevant contrast agents. Tf, reported as a surface modifier for iron oxide, can prolong the circulation time of materials in the bloodstream by modifying iron oxide. This modification enables multimodal imaging, targeted delivery to cancer cells, increased accumulation of contrast agents in target organs, and responsive release of contrast agents (Liu et al., 2013b; Zhi et al., 2020). Research on Tf-targeted nanocontrast agents primarily focuses on improving the precision of detection for small tumors and metastatic tumors. The primary emphasis lies in the detection of malignant tumors in the brain, aiming to enhance accuracy in this challenging context (Korotcov et al., 2010). Research has revealed that administering superparamagnetic iron oxide NPs to mice with C6 glioma can illustrate the benefits of targeted tumor cell delivery using Tf in comparison to non-targeted controls. Compared to non-targeted formulations, the signal intensity variation is more pronounced in mice with C6 glioma when Tf-targeted NPs are administered. Simultaneously, *in vitro* studies have also shown that this nanoparticle formulation increases the cellular uptake of contrast agents, corroborating research findings on cellular uptake and signal enhancement. Compared to non-targeted formulations, the signal intensity variation is more pronounced in mice with C6 glioma when Tf-targeted NPs are administered (Korkusuz et al., 2013). The current limitations of clinically relevant contrast agents include their inability to penetrate the BBB. The study found that, compared to nano carriers lacking Tf, Tf-bound NPs exhibit significant contrast enhancement in MRI within the brain (Anthony et al., 2011).

In recent years, research has found that positron emission tomography (PET) ligands based on TfR antibodies can be successfully used for brain imaging. The amyloid β (Ab) antibody mAb158, when radiolabeled and conjugated to a TfR antibody, serves as a PET ligand for the diagnosis and evaluation of Ab. The study found that PET imaging using this ligand in Alzheimer’s mouse models clearly displays Ab regions in the brain. The PET signal increases with the age of the subjects and correlates closely with Ab levels in the brain (Sehlin et al., 2016).

## 8 Challenges and future directions for current Tf-TfR1 drug delivery system

The targeted delivery of drugs through Tf-TfR1 drug delivery systems is an emerging alternative for cancer treatment. This drug delivery system has several advantages, including reducing systemic drug distribution, achieving high drug concentrations at the lesion, and realizing synergistic toxicity towards tumor cells. Despite such promising prospects, current drug delivery methods for cancer still face numerous limitations and challenges.

The inherent sensitivity of Tf to pH and enzymatic degradation imposes limitations on the drug delivery systems it mediates. This prompts the modification of Tf or the design and study of more stable analogs and new ligands to enhance binding affinity, prolong the half-life of drug circulation, and improve precise targeting of specific cells or tissues. After TfR is internalized, the endocytic vesicle enters an endosomal compartment, and during subsequent acidification, the decreased pH leads to a reduction in the affinity of Tf for iron, thereby inducing the release of iron (Skjorringe et al., 2015). This suggests that, for drugs binding to Tf, designing a pH-sensitive drug delivery system may be a relevant strategy. However, it is important to note that the impact of decreased pH on TfR goes beyond iron release. Crystal structure studies of TfR indicate significant conformational changes in the receptor protein structure during pH reduction. This may affect the binding of antibodies or other ligands to these locations (Steere et al., 2012).

Through pulse-chase experiments on a panel of different TfR antibodies using an *in vitro* model of the blood-brain barrier, it was observed that, despite having the same affinity, the efficiency and mode of crossing the blood-brain barrier varied among antibodies (Sade et al., 2014). This phenomenon was attributed to differences in the pH-sensitivity of the binding mode. The pH-insensitive variant exhibited strong binding and high uptake but was directed for degradation during intracellular sorting. In contrast, the pH-sensitive variant was associated with late endosomes and showed a higher degree of transcytosis. Therefore, by altering the ligand’s pH sensitivity, activation during endosomal acidification can be achieved, directing subsequent intracellular sorting toward transcytosis rather than degradation. One approach to modulating ligand pH sensitivity is to target it to regions of the TfR protein known to undergo conformational changes during endosomal acidification (Kim et al., 2016). pH-sensitive single-chain variable fragments were also designed as a feature of nanocarrier design, enabling nanoparticle release from the receptor-ligand complex and inducing transcytosis with subsequent distribution within the brain parenchyma (Clark and Davis, 2015).

Additionally, research has identified another significant limitation—the inability to efficiently bind a large quantity of therapeutic compounds with Tf (Karagiannis et al., 2006). This hinders the delivery of high-concentration drugs to target cells within the endocytic cycle mediated by Tf, consequently diminishing the intensity and efficiency of drug delivery. The inherent characteristics of tumors, such as complexity and heterogeneity, reduce the delivery efficiency of Tf-mediated drug delivery systems. Poor blood flow, stromal cell barriers, and dense extracellular matrix create a complex tumor microenvironment (TME) that interferes with tissue penetration of drug delivery systems, resulting in reduced delivery efficiency. Targeted drugs need to be covalently linked to nano-carriers to achieve specific targeting. There have been recent advancements in the design and development of nanoparticle. Nanocarriers generally exhibit good universality, with advantages such as high surface area, excellent biocompatibility, and ease of modification and encapsulation (Riccardi et al., 2021). The same nanocarrier can carry drugs of different properties by introducing them onto the surface or inside the nanocarrier through encapsulation, covalent binding, or physical adsorption (Rabiei et al., 2020). Therefore, by rationally designing nanocarriers, it is possible to overcome various shortcomings associated with direct drug administration, such as low brain penetration efficiency, poor targeting, high biological toxicity, and large drug doses (Teleanu et al., 2019). However, the translation from theoretical and laboratory research to clinically relevant drugs still face numerous limitations.

The safety and side effects of nanomaterials still require further investigation. The impact of the surface properties, particle size, and host material of nano-carriers on crossing the blood-brain barrier needs to be further assessed. Furthermore, the degradation and biocompatibility of nano-carriers are also research priorities that require significant attention. Many nano-materials are toxic to tissues, posing a risk of triggering the immune system. Moreover, since most targeted drugs aim at actively treating cancer, the addition of nano-materials increases the complexity of formulations, leading to elevated risks of toxicity and immunogenicity, while also raising production costs (Yusuf et al., 2023). When administered systemically, NPs are prone to be enveloped by serum albumin, immunoglobulins, and other blood components. This encapsulation may result in the substantial accumulation of NPs in the liver and spleen, with only a small fraction of the drugs actually reaching the tumor (Yan et al., 2005). To address this issue, studies have found that pairing with PEG during the preparation of NPs can prevent immune system detection and successfully avoid premature opsonization. PEG can also inhibit the binding of plasma proteins to the surface of NPs, thereby extending the circulation time of NPs in the body and enhancing their chances of reaching the disease site (Jain and Jain, 2008; Noble et al., 2014; Ramalho et al., 2022).

BBB limiting the effective transport of therapeutic drugs to the CNS. Moreover, the efficiency of brain-targeted delivery is low, and after intravenous administration, drugs are easily phagocytosed by the reticuloendothelial system, preventing the attainment of effective therapeutic drug concentrations in the brain, leading to suboptimal treatment efficacy.

Intranasal (IN) drug delivery, as a method for administering therapeutic agents for various medical conditions, has been extensively researched. Studies have found that IN drug delivery can bypass the blood-brain barrier, directly target the brain, and treat CNS disorders. The nasal route offers several advantages in the context of drug delivery for the treatment of glioblastoma, as it provides relatively easy access to the target area. The highly vascularized structure facilitates the absorption of drugs into the bloodstream, and it allows for lower medication doses by circumventing hepatic metabolism (Morales and Mousa, 2022; Wu et al., 2023). Research has found that IN delivery of liposomes loaded with monosialotetrahexosylganglioside (ganglioside GM1) can reach the tumor site through the lymphatic system. This delivery is accompanied by the use of near-infrared light (NIR) for photostimulation of lymphatic vessels, generating reactive oxygen species (ROS) and improving the anti-cancer effects (Semyachkina-Glushkovskaya et al., 2023). Another method to facilitate the transport of therapeutic agents across the blood-brain barrier is through the use of focused ultrasound (FUS). Research has found that the blood-brain barrier can be locally and temporarily disrupted by combining low-intensity focused ultrasound (LIFU) with microbubbles (Mitusova et al., 2022; Ramalho et al., 2022). Lin et al. (2016) utilized low-intensity focused ultrasound (LIFU) to induce the opening of the blood-brain barrier, allowing DOX-loaded cationic liposomes to be delivered to gliomas. This approach inhibited tumor growth while reducing side effects. The efficacy of this system was also confirmed in a murine model of resistant gliomas (Papachristodoulou et al., 2019). In recent years, TfR-targeting peptides have shown a trend as alternatives to antibodies due to their advantages of binding to TfR without competing with plasma Tf. The smaller size of TfR-targeting peptides provides an advantage over conventional antibodies and Tf molecules in traversing the blood-brain barrier (Sachdeva et al., 2019; Li et al., 2023). Therefore, surface modification of NPs with TfR-targeting peptides can optimize the performance of NPs, maintaining high receptor specificity and enhancing the precision of targeted delivery (Lakkadwala et al., 2019).

## 9 Conclusion

Iron homeostasis imbalance can lead to various diseases, including cardiovascular diseases, endocrine disorders, malignant tumors, etc., due to oxidative stress and endothelial cell damage. Targeting proteins associated with abnormal iron metabolism is essential for the management of a range of health conditions. The exploration of Tf has been ongoing since 1946, and to this day, our understanding of Tf has become more comprehensive. This includes its discovery, evolution, encoding, gene regulation, spatial configuration, iron ion transport, and the binding of TfR1. Subsequently, the biological functions of Tf and its applications in clinical therapy have further deepened the foundational research on this protein. Simultaneously, a potential therapeutic strategy has been recognized: utilizing Tf to sequester free iron, thereby delivering drugs into rapidly growing cells, activating the body’s immune cells, and preventing cell apoptosis. Research has found that Tf, as a protein abundant in plasma, is an ideal candidate for purification from plasma due to its crucial role in transporting iron and regulating iron metabolism in the body. The recombinant forms of Tf can be constructed by altering its metal binding sites or inserting peptide sequences, from its original spatial structure. Through this approach, it is possible to tailor drug delivery systems, offering the potential for designing more effective drug delivery systems. At the same time, the amalgamation of Tf with nanotechnology has catalyzed the creation of numerous innovative drugs and enhanced formulations, showcasing exceptional therapeutic effects.

Compared to traditional treatment methods, novel drug delivery systems exhibit excellent biocompatibility, enhancing drug stability while reducing toxic side effects. The targeting capability of drug delivery systems stands as a pivotal factor contributing to their extensive application prospects and clinical value. By designing delivery systems with specificity, precise transport of drugs or therapeutic genes to the site of pathology can be achieved.

This reduces toxic side effects on non-target tissues, and enhances treatment efficacy. This approach not only elevates local drug concentrations but also aids in minimizing side effects, thereby enhancing the overall quality of life for patients. However, creating drug delivery systems with substantial drug loading, non-toxicity, and high efficiency poses a notable challenge in design and synthesis. The continued advancement of drug delivery systems is poised to exert a profound influence on the realm of human healthcare. To achieve practical clinical applications, Tf stands as an ideal candidate molecule. Continuing in-depth and comprehensive research and development of Tf, along with its drug delivery systems, will provide more insights for clinical applications, as well as more improved therapeutic solutions for clinical treatment.
